# *Sarcocystis* Species Richness in Sheep and Goats from Lithuania

**DOI:** 10.3390/vetsci10080520

**Published:** 2023-08-11

**Authors:** Alina Marandykina-Prakienė, Dalius Butkauskas, Naglis Gudiškis, Evelina Juozaitytė-Ngugu, Dovilė Laisvūnė Bagdonaitė, Muza Kirjušina, Rafael Calero-Bernal, Petras Prakas

**Affiliations:** 1 Nature Research Centre, Akademijos 2, 08412 Vilnius, Lithuania; alina.prakiene@gamtc.lt (A.M.-P.); dalius.butkauskas@gamtc.lt (D.B.); naglis.gudiskis@gmail.com (N.G.); evelina.ngugu@gamtc.lt (E.J.-N.); bagdonaitedl@gmail.com (D.L.B.); 2 Institute of Life Sciences and Technology, Daugavpils University, Parādes Street 1A, 5401 Daugavpils, Latvia; muza.kirjusina@du.lv; 3 SALUVET Group, Animal Health Department, Complutense University, Ciudad Universitaria s/n, 28040 Madrid, Spain; r.calero@ucm.es

**Keywords:** *Sarcocystis*, small ruminants, intermediate host-specificity, *cox1*, phylogeny, microscopy

## Abstract

**Simple Summary:**

Protozoan parasites of the genus *Sarcocystis* are characterized by a mandatory two-host, prey–predator life cycle. Several *Sarcocystis* species are known to form macroscopic or microscopic sarcocysts in the muscle tissues of domestic sheep and goats. It has been long considered that *Sarcocystis* species parasitizing farm animals are specific to intermediate hosts. However, some studies have recently reported the unexpected detection of *Sarcocystis* species in animals that are not considered to be their canonical hosts. In the current investigation, muscle samples of sheep and goats from Lithuania were molecularly tested for species previously described in such hosts and for other non-canonical *Sarcocystis* spp. Based on DNA sequence analysis, along with canonical *Sarcocystis* species present in their respective hosts, non-canonical (atypical) species, such as *S. capracanis* and *S. morae*, were detected in sheep, while *S. arieticanis* and *S. tenella* were found in goats. Possible explanations of the obtained results are discussed.

**Abstract:**

Contradictory data is available on the intermediate host specificity of *Sarcocystis* spp. in farm animals. Therefore, the current work aimed at molecularly testing samples of sheep and goats reared in Lithuania to identify *Sarcocystis* species described in other intermediate hosts but suspected to be non-canonical parasites to these small ruminants. For this purpose, muscle samples from 47 domestic sheep and nine goats were examined. *Sarcocystis* species were identified using direct and nested PCR targeting *cox1* and sequencing of positive amplified products. Along with the detection of the canonical *Sarcocystis* spp. in their respective intermediate hosts, the DNA of *S*. *capracanis* and *S*. *morae* was detected in sheep, although these species were previously thought to be specific to goats and deer, respectively. In addition, DNA from *S*. *arieticanis* and *S*. *tenella* was found in goats, even though these two species were believed to be sheep-specific. Notably, under light microscopy, only sarcocysts of *S*. *capracanis* specific to goats were observed. Thus, future research on the life cycle and host-specificity of *Sarcocystis* spp. examined is warranted.

## 1. Introduction

Parasites of the genus *Sarcocystis* have a mandatory two-host life cycle that is usually rooted in an ecological prey–predator relationship. Typically, herbivores such as sheep (*Ovis aries*) and goats (*Capra hircus*), act as intermediate hosts, while carnivores, such as Felidae and Canidae, are definitive hosts. Specific intermediate hosts can become infected by ingesting food or water contaminated with *Sarcocystis* oocysts or sporocysts. Depending on the *Sarcocystis* spp., either microscopic or macroscopic tissue cysts (sarcocysts) will develop and allocate (especially) in the striated muscle of the intermediate host. When meat containing mature sarcocysts is consumed by a predator or scavenger, the parasite undergoes sexual reproduction and forms isosporoid oocysts in the intestines of the definitive host [1]. 

A significant number of reports of *Sarcocystis* spp. infection in sheep is available worldwide [2,3,4,5]. Comprehensive studies indicate that at least four *Sarcocystis* species, *S. arieticanis*, *S. tenella*, *S. gigantea*, and *S. medusiformis*, use sheep as intermediate hosts [6]. Both *S. arieticanis* and *S. tenella* form microscopic sarcocysts in the muscles of sheep and use canids as definitive hosts [1]. Meanwhile, *S. gigantea* and *S. medusiformis* form macroscopic sarcocysts in the muscles of sheep and use felids as definitive hosts [7]. Two additional species, *S. mihoensis* and *S. microps*, proposed to have dogs as the definitive host, have only been identified in two reports from Japan and China, respectively, and available molecular and biological details are scarce [8,9]. Usually, *Sarcocystis* infection is non-lethal to the ruminant intermediate host; nonetheless, the economic impact on profits associated with reduced production and carcass condemnation should be taken into consideration [1,2]. By contrast, significantly fewer reports on *Sarcocystis* spp. infecting domestic goats are available. Three different *Sarcocystis* species, *S. capracanis*, *S. hircicanis,* and *S. moulei*, are proposed to use goats as intermediate hosts. Two species, *S. capracanis* and *S. hircicanis*, form microscopic sarcocysts and use canids as definitive hosts, while *S. moulei* constitutes macroscopic sarcocysts and uses felids as definitive hosts (reviewed by [10]). However, most of the research has been conducted on *S. capracanis*, considered to be the most frequent and of most pathogenicity in goats [11]. To date, no zoonotic *Sarcocystis* spp. have been detected in small ruminants.

Recent studies indicate that the *18S* rRNA molecular marker has limited discriminatory power when investigating closely related *Sarcocystis* spp. infecting ungulates as intermediate hosts. In contrast, *cox1* was successfully used to identify *Sarcocystis* species present in sheep and goats [12,13,14]. Accurate species identification based on appropriate molecular markers and further sequencing confirmation procedures are key elements for a comprehensive investigation of the complex epidemiological scenario of the *Sarcocystis* genus. 

For a long time, it has been widely assumed that *Sarcocystis* spp. found in farm animals are strictly specific to the intermediate host [1]. However, *Sarcocystis* species have recently been increasingly reported in non-specific hosts. For instance, goat-specific *S. capracanis* has been reported in domestic sheep [15], while *S. moulei* that also utilizes goats as an intermediate host has been reported both in domestic sheep [16,17] and water buffalo (*Bubalus bubalis*) [18]. Moreover, sheep-specific *S. tenella* has been detected in wild goats (*Capra aegagrus*) [11,19]. Nevertheless, the outcomes of some of these findings are currently under discussion, and additional molecular and ultrastructural analyses are required [20]. 

In Lithuania, among farm animals, cattle (*Bos taurus*), horses (*Equus ferus caballus*), pigs (*Sus scrofa*), and sheep were previously examined for the presence of *Sarcocystis* spp. by means of morphological [21] and molecular methods [22,23]. Specifically, investigations carried out on sarcocysts excised from various analyzed muscles (oesophagus, diaphragm, and heart) of sheep by means of *cox1* sequence analysis revealed the occurrence of *S. arieticanis* and *S. tenella* [23]. By contrast, no previous studies have been conducted on the identification of *Sarcocystis* spp. in goats bred in the country. 

Given the contradictory data on the host-specificity of *Sarcocystis* species forming sarcocysts in sheep and goats, the present study aims at characterizing morphologically and genetically *Sarcocystis* sarcocysts that are present in the muscles of domestic goats, and to detect through robust *cox1* sequence analysis the potential presence of other non-canonical *Sarcocystis* spp. in Lithuanian sheep and goat muscle homogenates.

## 2. Materials and Methods

### 2.1. Sample Collection

Between 2019 and 2021, diaphragm, oesophagus, and heart samples were collected from 47 healthy domestic sheep following the slaughter process. Furthermore, three diaphragms, five hearts, and eight oesophagi were collected from healthy goats. The entire organs were obtained to examine the presence of *Sarcocystis* spp. infection. According to the Government system of animal registration, among the sheep, seven were under a year old, and 40 were adults. Among the goats, four were younger than one year, while the remaining four were older than two years. Sheep and goats were slaughtered for the purpose of meat consumption at the licensed slaughterhouse “Alantos agroservisas, UAB”, located in the village of Alanta, Molėtai district. The investigated sheep and goats were mainly raised in the eastern part of Lithuania. Muscle samples of slaughtered animals were taken by the company veterinarian and delivered to the Laboratory of Molecular Ecology, Nature Research Centre (Vilnius, Lithuania) for detailed morphological and molecular analysis of *Sarcocystis* spp. The transported muscle samples were stored frozen (at −20 °C) until further analysis. The general information about sheep and goats is known to the company’s veterinarian, and the farmers’ consents have been provided to the company. None of the animals were intentionally euthanized for the purpose of this research, and all sample collection procedures adhered to accepted animal welfare guidelines. 

### 2.2. Microscopic Examination for the Presence of Sarcocysts in Goat Tissues 

The collected tissue samples from goats were visually checked for macroscopic *Sarcocystis*-like structures. Subsequently, a morphological analysis of the microscopic sarcocysts was conducted using freshly squashed muscle samples. A microscopical examination was performed under a Nikon ECLIPSE 8oi light microscope (Nikon Corp., Tokyo, Japan). Sarcocysts excised from fresh muscle samples were preserved individually in separate tubes containing 96% of ethyl alcohol at −20 °C for the molecular examination.

### 2.3. Molecular Analysis of Sarcocysts Excised from Goat Tissues 

DNA extraction of sarcocysts (*n* = 2) was carried out with the GeneJET Genomic DNA Purification Kit (Thermo Fisher Scientific Baltics, Vilnius, Lithuania) in accordance with the manufacturer’s recommendations.

Partial *cox1* sequences were amplified by conventional PCR using forward SF1 [24] and reverse SsunR3 [20] primers. Each PCR reaction was carried out in a 25 μL mixture containing 12.5 μL of DreamTaq PCR Master Mix (Thermo Fisher Scientific Baltics, Vilnius, Lithuania), 5 μL of DNA template, 0.5 μM of both forward and reverse primers and nuclease-free water. The cycling conditions were the same as described [23] previously. The amplified products were visualized by horizontal 1% agarose gel electrophoresis. Positive samples were purified with exonuclease ExoI and phosphatase FastAP (Thermo Fisher Scientific Baltics, Vilnius, Lithuania).

Sequencing of the selected PCR samples was performed using a Big-Dye^®^Terminator v3.1 Cycle Sequencing Kit (Thermo Fisher Scientific Baltics, Vilnius, Lithuania) and a 3500 Genetic Analyzer (Applied Biosystems, Foster City, CA, USA), following the manufacturer’s specifications. The resulting *cox1* sequences of *Sarcocystis* spp. were compared with the most similar ones available in NCBI GenBank using the Nucleotide BLAST function (http://blast.ncbi.nlm.nih.gov/, accessed on 12 June 2023).

### 2.4. Acid-Pepsin Digestion of Muscle Samples from Sheep and Goats

Aiming for sensitive detection of *Sarcocystis* spp., muscle samples from 47 sheep (*n* = 141) and nine goats (*n* = 16) were subjected to identification of *Sarcocystis* spp. by combined acid-pepsin digestion and PCR techniques [23]. Artificial digestion was carried out by following a modified protocol that was initially designed for the isolation of *Toxoplasma gondii* from animal tissues [25]. Samples were prepared by cutting several pieces of tissue from different parts of the organ. Twenty-five g of each muscle sample were added to 150 mL of saline and blended. Further procedures of pepsin digestion were performed according to our previous study [23]. 

### 2.5. Molecular Detection of Specific Sarcocystis spp. in the Tissue Digests

All digested samples were subjected to the extraction of the genomic DNA using a PureLink Microbiome DNA Purification Kit (Invitrogen by Thermo Fisher Scientific, Waltham, MA, USA). For the specific detection of the canonical species that are present in domestic sheep (*S*. *arieticanis*, *S*. *gigantea*, *S*. *medusiformis*, *S*. *mihoensis*, and *S*. *tenella*) and goats (*S*. *capracanis*, *S*. *hircicanis*, and *S*. *moulei*) and to investigate their degree of unspecificity, conventional and nested PCR (nPCR) assays were employed (see below). In addition, given the previous reports of the wide host range of *S. morae* [26,27,28], specific tests were carried out. Primers used for direct and nPCR are compiled in Table 1. Due to the absence of available *cox1* data in the GenBank regarding *S. moulei* when the primers were designed, the *28S* rRNA gene was chosen as the molecular target for investigating this particular species of *Sarcocystis*. 

Positive DNA controls for *S*. *arieticanis*, *S*. *capracanis*, *S*. *morae*, and *S*. *tenella* were obtained from the individual sarcocysts acquired during our previous investigations [20,23,27]. The specificity of the primers was evaluated by testing them with DNA taken from sarcocysts of the particular *Sarcocystis* species being studied. Only the DNA from the targeted species produced amplicons of the expected sizes using the designed primers.

Each sample was subjected to conventional and nPCR assays. For both direct PCR and nPCR, the same conditions were employed as previously described [23]. Except for the annealing temperatures (53–61 °C), which were chosen according to the primer pair used for the reaction (Table 1), negative controls were employed as described in our earlier study [23]. 

### 2.6. Phylogenetic and Statistical Analysis

The *cox1* sequences of *Sarcocystis* spp. from sheep and goat tissues and from the individual sarcocysts of goat samples were deposited in GenBank with the accession numbers OR258580-OR258681. The obtained sequences were compared with those of the most closely related *Sarcocystis* spp. using Nucleotide BLAST (http://blast.ncbi.nlm.nih.gov/, accessed on 12 June 2023). For the phylogenetic analysis, sequences from this work were aligned with those gathered from the GenBank database by using the MUSCLE algorithm available in the MEGA7 software [29]. The final alignment consisted of 241 nucleotide positions and 29 taxa. The selection of the nucleotide evolutionary model (the Kimura 2-parameter + G) [30] and the construction of a phylogenetic tree under Bayesian inference were carried out by TOPALi v2.5 software [31]. For the analysis, general settings were as follows: 2 runs, 1,000,000 generations, 10 sample frequencies, and 25% burn-in. The phylogenetic tree was rooted in *S. truncata* from wild ungulates.

## 3. Results

### 3.1. Morphological Observations and Molecular Identification of Sarcocysts Present in Goat Tissues

**Figure 1:**
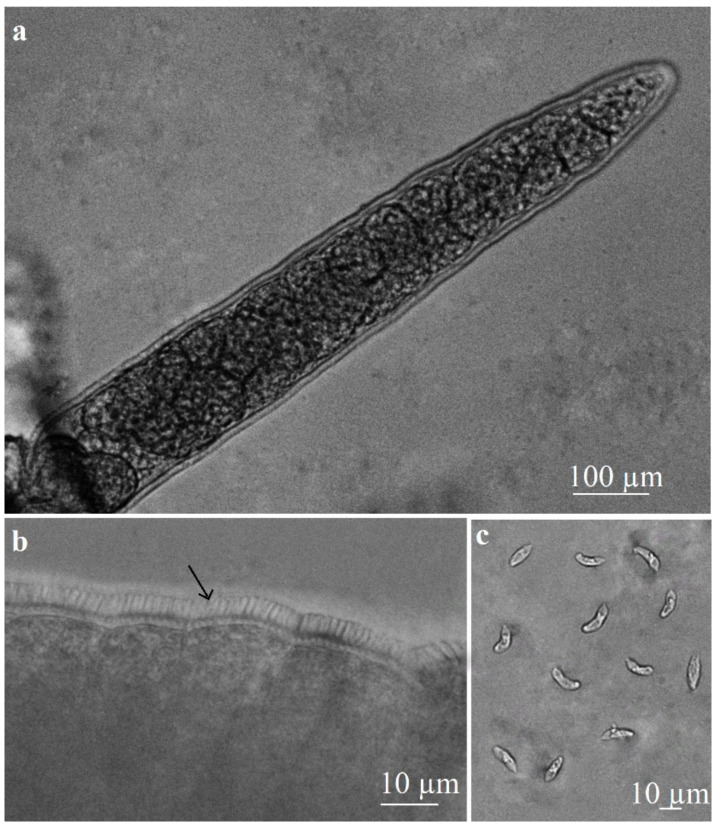
Light microscopy morphology of *S*. *capracanis* excised from the oesophagus of a goat. Fresh preparations. (**a**) An elongated, spindle-shaped fragment of sarcocyst. (**b**) A portion of sarcocyst wall with finger-like protrusions to which arrow is pointed. (**c**) Banana-shaped bradyzoites.

By fresh squeezing, *Sarcocystis*-like sarcocysts were found in a single oesophagus specimen. One type of microcyst similar to those of *S*. *capracanis* was observed under a light microscope. Sarcocysts were elongated and spindle-shaped, measuring 500–1310 × 65–150 µm (948 ± 237 × 109 ± 24 µm; *n* = 20) in size (Figure 1a). The sarcocyst wall was thick with radial striations and finger-like protrusions, which were 2.8–4.6 µm (3.9 ± 0.5 µm; *n* = 20) in length (Figure 1b). Cysts were septated, and their inner compartments were filled with elongated, banana-shaped bradyzoites measuring 11.2–15.9 × 2.5–4.7 µm (14.0 ± 1.2 × 3.7 ± 0.5 µm; *n* = 60) in size (Figure 1c). 

Two sarcocysts were isolated from the oesophagus of a single goat (ChLTC33st.1 and ChLTC33st.2) and genetically characterized within *cox1*. The obtained 894 bp sequences showed 99.1% identity compared with each other. In comparison to the sequences available in the GenBank, they shared 98.0–99.6% identity with those of *S*. *capracanis* and demonstrated 93.5–94.4% similarity to the sister species *S*. *tenella* of sheep.

### 3.2. Molecular Identification of Sarcocystis spp. in the Digested Samples of Sheep and Goats Using Species-Specific PCR 

Based on the species-specific PCRs using primers targeting *cox1*, two species, *S*. *arieticanis* and *S*. *tenella*, were identified in sheep tissues, and *S*. *capracanis* was confirmed in the muscles of goats by both conventional and nPCR (Table 2). By contrast, attempts to amplify *S*. *gigantea*, *S*. *medusiformis*, and *S*. *mihoensis* in samples of sheep and *S. hircicanis* and *S. moulei* in samples of goats using the designed primers did not yield any positive results. Apart from *Sarcocystis* species specific to their respective intermediate hosts, *cox1* fragments of *S*. *capracanis*, *S*. *morae*, and an undescribed genetic variant, *Sarcocystis* sp. OaLT1, as well as those of *S*. *arieticanis* and *S*. *tenella,* were amplified in sheep and goats, respectively. In the cases of all molecularly confirmed *Sarcocystis* spp., the intraspecific variability values did not overlap with those of interspecific variability. In addition, similar values of intraspecific variability were obtained when comparing sequences of the same species from both sheep and goats. For instance, the *cox1* sequences of *S*. *tenella* from sheep and goats demonstrated 96.0–100% and 95.7–100% similarity, respectively, compared to sequences of this species deposited in NCBI GenBank.

In addition to the previously genetically characterized *Sarcocystis* species, an undescribed *Sarcocystis* sp. OaLT1 genetic variant was observed in the diaphragm muscles of two sheep. Two 100% identical 241 bp sequences of *Sarcocystis* sp. OaLT1 were obtained using direct PCR and primers designed for the identification of *S*. *morae* (Table 1). These sequences displayed 80.3–83.8% similarity to *S*. *tenella*, 78.6–81.9% similarity to *S*. *capracanis*, and 80.4% similarity to *S*. *heydorni* of cattle (Table 2). 

**Figure 2:**
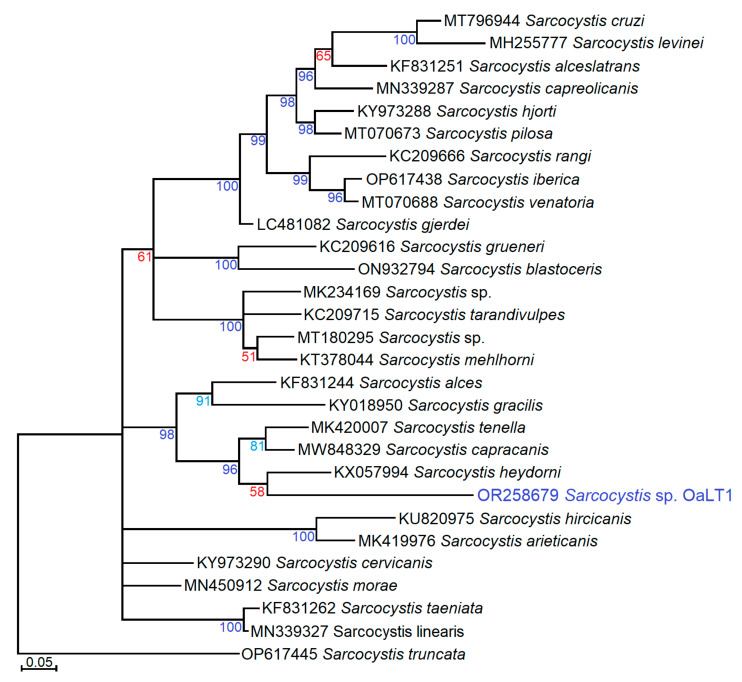
The phylogenetic tree of some *Sarcocystis* spp. using *cox1* sequences and Bayesian methods, displaying the placement of new variant *Sarcocystis* sp. OaLT1 from sheep detected in the present study. The tree was rooted on *S*. *truncata*. Percentage posterior probability values higher than 50, 70, and 95 were presented in red, turquoise, and indigo, respectively.

Based on a short fragment of *cox1*, *Sarcocystis* sp. OaLT1 clustered together with *Sarcocystis* spp. using ungulates and canids as their intermediate hosts and definitive hosts, respectively (Figure 2). The exact phylogenetic position of *Sarcocystis* sp. OaLT1 was not resolved because a low posterior probability value (58) was obtained for the grouping of the newly identified *Sarcocystis* sp. OaLT1 variant with *S*. *heydorni* of cattle. However, the phylogenetic analysis showed close relationships of *Sarcocystis* sp. OaLT1 and *Sarcocystis* spp. parasitizing sheep (*S*. *tenella*), goat (*S*. *capracanis*), cattle (*S*. *heydorni*) [32], moose (*Alces alces*) (*S*. *alces*) [33] and roe deer (*Capreolus capreolus*) (*S*. *gracilis*) [34].

### 3.3. The Detection Rates of Sarcocystis spp. in Sheep Tissues by Direct and Nested PCR 

The occurrence of *S*. *arieticanis* and *S*. *tenella* in the diaphragm, heart, and oesophagus of sheep ranged from 83.0% to 100% and from 10.6% to 59.6% by nPCR and direct PCR, respectively. At the same time, the occurrence of *S*. *capracanis* and *S*. *morae* in three types of sheep muscles was in the range of 10.6–42.6% and 10.6–31.9%, respectively (Table 3).

Positive samples indicating the presence of *S. capracanis* and *S. morae* were validated through the sequencing of positive amplicons obtained from both conventional PCR and nPCR. In the cases of *S. arieticanis* and *S. tenella*, two samples from the oesophagus, diaphragm, and heart were obtained through conventional PCR and subjected to sequencing. The same sequencing procedure was applied to the positive amplicons derived from the nPCR of the respective *Sarcocystis* species under investigation.

## 4. Discussion

The present study revealed the first identification of *Sarcocystis* species in goats raised in Lithuania. Based on molecular examination, *S. capracanis* was observed in three out of nine (33.3%) goats tested. In addition, *S. arieticanis* was detected in a single goat (11.15%), and *S. tenella* was found in four (44.4%) individuals (Table 2). Both species are known to use domestic sheep as intermediate hosts [1]. In total, results of molecular analyses showed that 8/9 (88.9%) of goats were infected with *Sarcocystis* spp. Notably, goats are infrequently reared for meat in Lithuania, and the number of goats in the country is almost 10 times lower than the number of sheep (https://osp.stat.gov.lt/lietuvos-aplinka-zemes-ukis-ir-energetika-2022/zemes-ukis/gyvulininkyste, accessed on 7 July 2023).

Studies on *Sarcocystis* spp. in goats have been mostly carried out in Asia and Africa. In Kunming (China), 174 of 225 (77.3%) goats were confirmed to be infected with *Sarcocystis* spp. using *cox1* region. The prevailing *S. capracanis* was detected in 74.6% of goats, and *S. hircicanis* was found in 33.3% of individuals [35]. Meanwhile, it was reported that the frequency of microscopic *Sarcocystis* in Saudi Arabia was lower (42.9%; 36/84 goats), as only *S. capracanis* was distinguished by *cox1* [14]. Only *S. capracanis* was identified in Malaysian goats by PCR targeting *18S* rRNA, revealing a level of infection of around 90.5% [36]. Other surveys conducted exclusively by light microscopy examination reported occurrences in Egypt, Iraq, and Iran ranging from 79.4–100.0% [37,38,39,40,41,42]. In general, given the small sample size of goats collected in Lithuania, reliable conclusions on the infection rates of *Sarcocystis* spp. are difficult to be drawn.

As commented above, an important number of recent studies suggest that the intermediate host-specificity of several *Sarcocystis* species may not be as strict as previously believed [1]. The reliability of the findings of some studies [18,43] is in doubt due to the absence of DNA sequencing data in the GenBank database. A report originating from Egypt indicated that *S. hjorti*, a parasite known to infect cervids [44], was found in cattle [43]. Nevertheless, according to the presented data, the sequence similarity only reaches 96% when compared to other *S. hjorti* sequences, suggesting that it is likely an undescribed *Sarcocystis* sp. rather than *S. hjorti* [43]. There are a couple of studies about *S. gigantea*, a species that typically parasitizes sheep [7], infecting cattle [45] or horses [46]. While the obtained *18S* rRNA sequences exhibited high similarities to *S. medusiformis* and *S. moulei*, they consistently displayed distinguishable differences from *Sarcocystis* species identified in cattle and horses (e.g., absence of secondary cyst wall). There is also an investigation showing that *S. cruzi*, which typically infects cattle, was detected based on *18S* rRNA in another bovid species, the wood bison (*Bison bison athabascae*) [47]. Regardless, there is a need to enrich previously described data on *18S* rRNA with additional investigation on other genetic regions, such as the *cox1* gene, as it possesses significant variability and serves as the most accurate method to differentiate taxonomically close *Sarcocystis* species [48].

Some existing reports mentioned that *S. tenella* and *S. capracanis* can parasitize both sheep and goats [35,49]. Furthermore, *S. tenella* has been detected in chamois (*Rupicapra rupicapra tatrica*) [19], and *S. tenella* and *S. capracanis* have been identified in Barbary sheep (*Ammotragus lervia*) [50]. However, both host species do not belong to the *Ovis* or *Capra* genera, thus raising questions about *Sarcocystis* species-specificity for their intermediate host. 

During this study, DNA of non-specific *Sarcocystis* spp. has been detected in heart, oesophagus, and diaphragm samples from sheep and goats (Table 2). To the best of our knowledge, this study represents the first detection of *S. morae* DNA in sheep as well as the first confirmation of *S. arieticanis* DNA in goats. Even though parasite DNA was detected in non-canonical hosts, no sarcocysts of the aforementioned *Sarcocystis* species have been found in any of the muscle samples. 

Several hypotheses arise herein to explain the results of the current study. The first and simplest explanation could be the contamination of the laboratory equipment and environment. However, such an explanation is unlikely for the following reasons: (i) the agarose gel of all negative controls remained clean; (ii) not all samples were positive for atypical/unexpected species; (iii) the genetic identity of all positive samples for atypical species was confirmed by the Sanger sequencing method showing multiple single peaks in each chromatogram; (iv) the obtained sequences of non-host-specific *Sarcocystis* species demonstrated intraspecific variation. The second hypothesis raised suggests that *S. capracanis* and *S. morae* form microscopic sarcocysts in the muscle tissues of sheep, while *S. arieticanis* and *S. tenella* form sarcocysts in the muscle tissues of goats. However, the load of sarcocysts of non-specific *Sarcocystis* species is negligible compared to the load of sarcocysts of the predominant typical species. A similar case has already been described when DNA of *S. hominis* was detected in digested muscle tissues of 14/102 cattle, but sarcocysts of this species specific to cattle were not found in diaphragm samples [22]. Real-time PCR would help to answer the question of the relative quantities of the identified species in the test samples [48]. In sum, the findings of the present study indicate that there are still many unanswered questions about the life cycle and host-specificity of *Sarcocystis* species infecting farm animals.

## 5. Conclusions

Microscopical and molecular identification of *S*. *capracanis* in excised sarcocysts from goats constitutes the first report of *Sarcocystis* spp. infection in such a host in Lithuania. Species-specific PCR tests allowed the detection of two non-typical *Sarcocystis* species in sheep, *S*. *capracanis* and *S*. *morae*, in the muscles of sheep, and two non-typical goat *Sarcocystis* species, *S*. *arieticanis* and *S*. *tenella*, in the muscles of goats. Additional molecular data showed the presence of a new *Sarcocystis* genetic variant (*Sarcocystis* sp. OaLT1) with 78.6–83.8% similarity to the closest *Sarcocystis* species cycling in ungulates as intermediate hosts in the muscle samples of two sheep.

Evidence of a lower intermediate host-specificity identified here warrants the need for further investigations, including additional effort on direct examination for individual sarcocysts and, when possible, experimental infections to demonstrate such cross-infections.

## Figures and Tables

**Table 1:** List of PCR primers, their sequences, annealing temperatures, predicted size of amplicons, and target genes.

*Sarcocystis* Species	Primer Name	Orientation	Primer Sequence	Ta (°C)	Fragment Size (bp)	Molecular Target
*S. arieticanis*	SF1 ^1^	Forward	ATGGCGTACAACAATCATAAAGAA	53	913	*cox1*
	SsunR3 ^2^	Reverse	CCGTTGGWATGGCRATCAT			
	**Arieticanis7F ^3^**	Forward	TAATTTCCTCGGTACTGTACTGTTTG	61	290	
	**Arieticanis7R ^3^**	Reverse	TACTTACGCATTGCGATATTACG			
*S. gigantea*	V2gig1 ^2^	Forward	GCACTTCGAGCATTCTTGG	57	548	
	V2gig2 ^4^	Reverse	ATCTACATCCACCGTAGGAACCTTA			
	V2gig3 ^4^	Forward	CAGCAAGTACCAAGTTCTGTACGTC	62	322	
	V2gig4 ^2^	Reverse	GGTGCCGAGTACCGAGATACAT			
*S. medusiformis*	V2medu1 ^2^	Forward	TTAATGGCATATCGTACTACCTATTG	56	729	
	V2medu2 ^2^	Reverse	CCCATGCATCAACCTCCAG			
	V2medu3 ^2^	Forward	GTATCCTGGGGGCCATTAACTT	61	389	
	V2medu4 ^2^	Reverse	CCAAACCAGTGTTCCGAGTATTG			
*S. mihoensis*	V2miho1 ^2^	Forward	ATCTTTACACTGCACGGTTTGTTT	60	844	
	V2miho2 ^2^	Reverse	AGTCGTTATGTCGGAAGTCAACAG			
	V2miho3 ^2^	Forward	GATGTTACCTCGGGTAAATGCTCTT	60	526	
	V2miho4 ^2^	Reverse	AAAAACATGTCTAGCTCCTAACACC			
*S. tenella*	SF1 ^1^	Forward	ATGGCGTACAACAATCATAAAGAA	53	913	
	SsunR3 ^2^	Reverse	CCGTTGGWATGGCRATCAT			
	**Tenella8F ^3^**	Forward	ATACCGCTCTACGCTGGATCTAC	59	421	
	**Tenella8R ^3^**	Reverse	AACCATCGTACAATCCAAAACTAAA			
*S. capracanis*	VocaF1 ^3^	Forward	GTAAACTTCCTGGGTACTGTGCTGT	60	531	
	VocaR1 ^3^	Reverse	CCAGTAATCCGCTGTCAAGATAC			
	**V2ca3 ^3^**	Forward	ATACCGATCTTTACGGGAGCAGTA	58	330	
	**V2ca4 ^3^**	Reverse	GGTCACCGCAGAGAAGTACGAT			
*S. hircicanis*	V2hirici1 ^3^	Forward	CCGTAGATGCCATGGGTACTT	61	868	
	V2hirici2 ^3^	Reverse	GTAGATATCCAGTGACGTGGTGAG			
	V2hirici3 ^4^	Forward	GCCTGGGTATTCTAGGACTGAGTAG	62	354	
	V2hirici4 ^4^	Reverse	CGAAAACTGCTCTACCGCTCA			
*S. morae*	SF1 ^1^	Forward	ATGGCGTACAACAATCATAAAGAA	53	913	
	SsunR3 ^2^	Reverse	CCGTTGGWATGGCRATCAT			
	**V2mor1 ^3^**	Forward	GTGTGCTTGGATCGGTCAAC	57	332	
	**V2mor2 ^3^**	Reverse	GCCGAATACCGGCTTACTTC			
*S. moulei*	V2moul1 ^3^	Forward	GGAGATTCTTGTTGAGTGGGTCT	54	790	*28S* rRNA
	V2moul2 ^3^	Reverse	GCAAAGCATAATATTTTCTAACGAT			
	V2moul3 ^4^	Forward	GTGAATGCCCTATGTTGTTGAG	59	496	
	V2moul4 ^4^	Reverse	ATATGTGAGAGTGTAGCCCGAAGA			

^1^ [24], ^2^ [23], **^3^** primer combinations designed during the present study, ^4^ [20]. Ta: annealing temperature; primers used for a direct PCR in the present study are in boldface.

**Table 2:** Identification of *Sarcocystis* spp. detected by conventional and nPCR in tissues from domestic sheep and goats and comparison of genetic variability.

Species	Host	Information of Sequences			Sequence Similarity, %	
		*n*	Length (bp)	GenBank Acc. No.	Comparing Sequences of the Same Species	Comparing Obtained Sequences with Those of Most Closely Related Species
*S. tenella*	Sheep	12	373	OR258580-OR258591	96.0–100	91.7–93.8 *S*. *capracanis*
***S. tenella***	Goat	5	373	OR258594-OR258598	95.7–100	91.4–93.8 *S*. *capracanis*
*S. arieticanis*	Sheep	12	241	OR258599-OR258604, OR258607-OR258612	96.9–100 *	84.7–86.7 *S*. *hircicanis*
***S. arieticanis***	Goat	1	241	OR258615	97.5–100 *	85.5–86.3 *S*. *hircicanis*
*S. capracanis*	Goat	3	284	OR258616-OR258618	97.2–99.7	90.4–93.7 *S*. *tenella*
***S. capracanis***	Sheep	27	284	OR258621-OR258641, OR258647-OR258652	96.8–100	90.5–93.0 *S*. *tenella*
***S. morae***	Sheep	22	292	OR258655-OR258670, OR258672-OR258677	95.6–100	82.9–85.3 *S*. *cervicanis*
***Sarcocystis*** **sp. OaLT1**	Sheep	2	241	OR258679-OR258680	No GenBank records	80.3–83.8 *S*. *tenella* 78.6–81.9 *S*. *capracanis* 80.4 *S*. *heydorni*

*Sarcocystis* species previously not identified in certain intermediate hosts are highlighted in bold. *n*—number of sequences. * Excluding two sequences MH413047–MH413048 of *S*. *arieticanis* from Egypt, the comparison in the present study obtained sequences with the above-mentioned sequences displayed only 90.5–92.5% similarity.

**Table 3:** The distribution of *Sarcocystis* spp. in different organs of sheep determined by nPCR and direct PCR. Values in cells indicate the number of positive samples, and values in brackets show the percentage ratio.

Species	Type of Sample					
	Diaphragm		Heart		Oesophagus	
	Nested	Direct	Nested	Direct	Nested	Direct
*S. arieticanis*	44 (93.6)	28 (59.6)	39 (83.0)	5 (10.6)	39 (83.0)	9 (19.1)
*S. tenella*	47 (100)	18 (38.3)	45 (95.7)	18 (38.3)	40 (85.1)	16 (34.0)
*S. capracanis*	20 (42.6)	10 (21.3)	11 (23.4)	5 (10.6)	13 (27.7)	6 (12.8)
*S. morae*	15 (31.9)	5 (10.6)	8 (17.0)	6 (12.8)	10 (21.3)	5 (10.6)

## Data Availability

The *cox1* sequences of *S*. *arieticanis*, *S*. *capracanis*, *S*. *morae*, *S*. *tenella* and *Sarcocystis* sp. OaLT1 were submitted to the GenBank database under the accession numbers OR258580-OR258681.
